# Insulin Intensification and Simplification Strategies in Patients with Type 2 Diabetes and Overweight or Obesity: A Scoping Review

**DOI:** 10.3390/ijms27209050

**Published:** 2026-10-12

**Authors:** Chew Huey Chua, Wei Lim Chong, Hasniza Zaman Huri

**Affiliations:** 1 Department of Clinical Pharmacy and Pharmacy Practice, Faculty of Pharmacy, Universiti Malaya, Kuala Lumpur 50603, Malaysia; cch1347@gmail.com (C.H.C.); williamchongwl@um.edu.my (W.L.C.); 2 Precision Medicine and Omics Centre (PrOmiC), Faculty of Pharmacy, Universiti Malaya, Kuala Lumpur 50603, Malaysia

**Keywords:** type 2 diabetes mellitus, insulin, intensification, simplification, overweight, obesity

## Abstract

Overweight and obesity complicate insulin treatment in adults with type 2 diabetes mellitus (T2DM), requiring consideration of glycaemic control, insulin requirements, body weight, and treatment burden. This scoping review mapped insulin treatment modification (ITM) strategies, synthesised their reported outcomes, and identified evidence gaps among insulin-treated adults with T2DM and overweight or obesity. Five electronic databases were searched following Preferred Reporting Items for Systematic Reviews and Meta-Analyses extension for Scoping Reviews (PRISMA-ScR) and updated Joanna Briggs Institute methodological guidance. Thirty-three studies were classified into four ITM domains: add-on therapy to insulin, switching to advanced insulin formulations, simplifying complex insulin regimens with adjunctive therapy, and switching to fixed-ratio combinations. Intensification was generally studied in patients with higher baseline glycated haemoglobin or insulin requirements, whereas simplification was more often evaluated in those receiving complex insulin regimens. Insulin-dominant intensification generally increased total daily insulin dose, while intensification incorporating Glucagon-Like Peptide-1 Receptor Agonists (GLP-1RAs) or Sodium-Glucose Cotransporter-2 Inhibitors (SGLT2i) commonly reduced insulin requirements and body weight. Simplification generally reduced insulin requirements and/or injection burden while maintaining or improving glycaemic control, with weight reduction frequently reported. Direct comparisons and obesity-specific subgroup analyses were limited. Intensification and simplification should therefore be considered complementary rather than opposing approaches, with outcomes influenced by therapeutic components and patient characteristics.

## 1. Introduction

The global burden of type 2 diabetes mellitus (T2DM) continues to rise alongside the obesity epidemic. Between 2021 and 2024, the number of adults living with T2DM in-creased from 509 million to approximately 550 million [1,2]. By 2022, obesity affected more than 1 billion individuals worldwide [3,4]. Nearly half of patients with T2DM have coexisting obesity, which is strongly associated with excess adiposity, impaired insulin sensitivity, and suboptimal glycaemic control [5,6]. However, body mass index (BMI) alone may underestimate obesity-related metabolic risk because it does not capture body fat distribution or underlying metabolic dysfunction. Individuals with similar BMI values may therefore differ substantially in metabolic phenotypes and treatment needs.

These limitations have prompted a shift toward more clinically grounded understanding of obesity. The Lancet Commission recently proposed the concept of clinical obesity, which combines anthropometric measures with obesity-related organ dysfunction to identify individuals who may benefit from targeted medical intervention [5]. In patients with T2DM, excess adiposity can contribute to systemic inflammation, hyperinsulinemia and chronic hyperglycaemia, thereby worsening insulin resistance [7,8]. As a result, overweight or obese patients with T2DM may require higher insulin doses or additional insulin-sensitising therapies to achieve glycaemic targets [9,10]. Insulin treatment itself may also promote further weight gain, adding to the long-term difficulty of diabetes management [11,12].

Despite newer glucose-lowering therapies, insulin remains indispensable in T2DM management. Progressive beta cell (β-cell) dysfunction means that many patients eventually require insulin, regardless of their initial treatment approach [13,14]. As β-cell function declines, insulin secretion becomes insufficient to compensate for insulin resistance, leading to persistent hyperglycaemia and an increasing need for exogenous insulin [13,14,15]. Hyperglycaemia can further impair β-cell function through glucose toxicity [15] and promote advanced glycation end-product formation, oxidative stress and inflammation, contributing to diabetes-associated tissue injury [16]. For overweight or obese patients already receiving insulin, treatment modification is therefore a common clinical challenge. These decisions are complicated by insulin resistance, weight gain, treatment burden, variation in insulin regimens, and heterogeneity in clinical and metabolic characteristics [9,11]. As obesity becomes more prevalent among insulin-treated adults with T2DM, understanding how insulin treatment is modified in practice has become increasingly important [9,17].

Insulin treatment modification (ITM) in this population generally follows two broad strategies: intensification and simplification [9,12,17]. Intensification aims to improve glycaemic control by increasing insulin dose, advancing to more complex insulin regimens, or adding adjunctive glucose-lowering therapies. Simplification aims to reduce treatment complexity and burden while maintaining glycaemic targets, often by lowering insulin dosage, reducing injection frequency, or switching to alternative therapeutic combinations [9,11]. In clinical practises, clinicians may use intensification, simplification or both, depending on glycaemic status, treatment burden, adiposity, insulin resistance, patient preference, and metabolic phenotypes [18,19,20].

Evidence-based guidance on insulin treatment modification specifically for overweight or obese adults with T2DM remains limited. Existing recommendations often emphasise the addition of weight-reducing non-insulin agents, but provide less detail on how insulin regimens themselves should be modified [12]. Moreover, intensification and simplification strategies have often been studied separately. Limited synthesis is available on when, why, and under what circumstances one approach may be favoured over another in this population. Clinicians therefore lack a consolidated view of insulin modification strategies, their clinical indications, and their reported outcomes when obesity complicates glycaemic management. To address this gap, this scoping review mapped the range of ITM strategies reported in this population, classified them into four regimen modification categories, summarised their reported clinical impacts, and identified evidence gaps for future research and clinical practice.

## 2. Materials and Methods

### 2.1. Study Design and Registration

This study was designed as a scoping review to map the main concepts of ITM strategies used in overweight and obese adults with T2DM. This review aimed to explore insulin simplification strategies as a future direction in insulin-related research, as no comprehensive reviews had synthesised the evidence specifically for overweight or obese adults. A scoping review was considered appropriate, as this topic remains underexplored despite its clinical importance. This scoping review was conducted in accordance with the Preferred Reporting Items for Systematic Reviews and Meta-Analyses extension for Scoping Reviews (PRISMA-SCR) checklists and the updated Joanna Briggs Institute (JBI) methodological guidance for scoping reviews [21,22]. The PCC (Population, Concept and Context) framework was used to define the scope of the review according to JBI guidance as presented in Table 1 [22]. The review write-up was then presented according to the PRISMA-SCR checklists.

Studies were eligible for inclusion if they met the following criteria: (a) peer-reviewed journal articles published in English between 2021 and March 2026; (b) primary quantitative studies involving participants with T2DM who underwent ITM and reported its impacts; and (c) secondary studies, including post hoc analyses, that met the inclusion criteria. The selected time window focused the review on contemporary evidence and recent developments in insulin treatment modification.

### 2.2. Research Question

1. What baseline characteristics were reported among overweight or obese patients with T2DM who underwent ITM?
2. What were the focus and trends of global studies on ITM among overweight and obese T2DM patients?
3. What ITM strategies were used, and what impacts were reported among overweight or obese patients with T2DM?

### 2.3. Search Strategy

A literature search was performed in PubMed, Scopus, COCHRANE, CINAHL Complete (via EBSCOhost) and MEDLINE (via OVID). The search strategy focused on ITM strategies and insulin regimens among T2DM patients across all care settings. We used a three-step search strategy based on JBI guidelines. First, we conducted a preliminary PubMed search to identify relevant journal articles. Second, we used keywords from the titles and abstracts, along with relevant Medical Subject Headings (MeSH) terms, to refine the search strategy for each database. Third, we manually screened the reference lists of included articles to identify additional relevant studies. The search strategy focused on four main concepts: “type 2 diabetes mellitus”, “insulin treatment modification”, “insulin regimen”, and “overweight or obesity”. We adapted both keywords and MeSH terms for each database. The full search strategies are provided in Supplementary Tables S1–S5.

### 2.4. Selection of Data

Records identified in electronic databases were imported into EndNote 2025 for duplicate removal. Non-English-language records were also removed at this stage. Two reviewers then independently screened the titles and abstracts of the retrieved records against the inclusion and exclusion criteria using Rayyan web tool (https://www.rayyan.ai/, accessed on 9 September 2026). Disagreements were resolved through discussion. Next, the full texts of potentially eligible articles were obtained, screened and assessed against the eligibility criteria. We excluded full-text articles that did not meet the criteria, and recorded the reasons.

### 2.5. Data Extraction

Data were charted using a standardised form developed in line with the variables specified for this review [23]. The charting form was revised and refined during data extraction. The form captured baseline characteristics of the study population, classification of overweight or obesity, ITM strategies, the insulin regimen used, a summary of findings, and the author and year of publication. The summary of findings reported the impacts of ITM strategies, including glycated haemoglobin (HbA1c), body weight, insulin total daily dose (TDD), hypoglycaemia risk, and treatment satisfaction or quality of life (QOL).

### 2.6. Data Analysis and Presentation

We summarised the extracted data into four themes derived from the research questions. Theme 1 examined global research on ITM, including the focus and trends involving overweight or obese T2DM patients. Theme 2 described the baseline profiles across modification strategies. Theme 3 and 4 summarised ITM strategies used and their reported impacts in this population. The operational definitions in Table 2 guided data extraction to ensure consistent classification. Intensification and simplification were not mutually exclusive: a treatment modification could meet both definitions when glucose-lowering treatment was escalated while insulin-regimen complexity was reduced. For example, adding a GLP-1RA or SGLT2i could constitute intensification while also simplifying the insulin regimen if it reduced injection frequency, the number of separately administered treatment components or administration complexity. Switching insulin formulations or delivery systems was classified as intensification only when it constituted treatment escalation to enhance glucose-lowering capacity. Switching to an FRC was classified as simplification only when it reduced regimen complexity relative to the previous treatment. Changes in total daily insulin dose alone did not determine classification. We presented findings in tables and visualised them using graphs generated in R version 4.5.2 to provide a comprehensive overview of the evidence [23].

## 3. Result

### 3.1. Search Result

The search identified 2956 records from five electronic databases. After title, abstract, and full-text screening, 33 studies were included. Figure 1 presents the study selection flow diagram. The included studies reported baseline age, T2DM duration, BMI, HbA1c, insulin TDD, study design, sample size, and follow-up duration. Their study designs included interventional studies such as randomised or non-randomised controlled trials, observational studies such as cross-sectional or cohort studies, and post hoc analyses. In this review, most intensification strategy studies were conducted through clinical trials, whereas observational study designs were more common among simplification studies. Moreover, several included studies reported subgroup analyses using post hoc analyses [24,25,26,27,28]. Most included studies had sample sizes of 101–500 participants, and more than half had follow-up durations of 5.5–7.5 months. The majority of studies measured outcomes at 6 months, but 23.8% of simplification strategy studies reported outcomes beyond 12 months (Table 3).

### 3.2. Theme 1: Geographical Distribution and Publication Trends

The included studies were conducted across multiple countries and regions. The largest number of studies came from the United States (US), Japan and Italy (Figure 2a). Both intensification and simplification strategies were investigated in these countries, although simplification studies were more frequent in most settings. China was an exception, with included studies mainly focused on intensification strategies or on both approaches within the same study.

Multinational studies generally evaluated intensification strategies and often had larger sample sizes. These included three randomised controlled trials (RCTs) and one post-hoc analysis. Across Asia-Pacific countries, including China, Japan, Taiwan, South Korea, Thailand and Australia, both intensification and simplification strategies were represented. In Western populations, simplification strategies were more commonly studied (Figure 2b).

Publication patterns also varied by strategy (Figure 2c). More than 60% of included studies published between 2021 and early 2026 examined simplification strategies, although no simplification studies were identified in 2022. By early 2026, all included studies focused on simplification strategies. Intensification strategies accounted for a smaller proportion of studies in 2023, 2024 and 2025.

### 3.3. Theme 2: Baseline Profiles Across Modification Strategies

Table 3 summarises the included studies’ characteristics by insulin treatment category, while Table 4 summarises baseline characteristics, insulin regimens, and key findings for all included studies classified under insulin intensification or simplification strategies. Of the 33 unique studies, 12 evaluated intensification only, 20 evaluated simplification only, and one evaluated both [29]. Consequently, 13 studies contributed to the intensification category and 21 to the simplification category. These categories were not mutually exclusive, with one study contributing to both. In Table 4, distinct regimens evaluated within the same study and treatment category were grouped under a single study entry [28,30,31,32]. Each study was counted once within each applicable category and once in the overall total, yielding 34 study–approach entries from 33 unique studies. As described in Table 4, intensification studies commonly included participants with higher baseline HbA1c, higher insulin requirements and longer T2DM duration. In these studies, most baseline HbA1c values were at least 8%.

In contrast, simplification studies included a wider range of glycaemic profiles. Baseline HbA1c levels varied across studies, with some enrolling participants with relatively stable glycaemic control and HbA1c levels close to 6.5%, while others included patients with HbA1c above 9% [33,34,35]. Additionally, simplification studies typically selected patients with lower insulin requirements, shorter T2DM duration and obesity. These approaches were commonly applied to patients receiving complex insulin regimens, especially basal-bolus or premixed insulin. Studies evaluating FRC approaches often require participants to have preserved β-cell function, as assessed by baseline C-peptide levels [35,36,37] (Table 3 and Table 4).

### 3.4. Theme 3: Insulin Treatment Modification Strategies

Four ITM strategy domains were identified from the included studies: add-on therapy to insulin, switching to advanced insulin formulations, simplification of complex insulin regimen with adjunct therapy, and switching to FRCs (Table 5). Classification of these strategies followed the operational definitions provided in Section 2.6 and Table 2. These approaches may overlap, as pharmacological intensification with adjunctive therapy can simultaneously simplify the insulin regimen.

### 3.5. Theme 4: Impacts of Strategies on Overweight and Obese T2DM Patients

The included studies reported clinical and treatment-burden outcomes, including HbA1c, body weight, insulin TDD, hypoglycaemia risk, injection burden, quality of life (QOL) or treatment satisfaction, and gastrointestinal adverse events. Figure 3 summarises the reported outcome patterns across intensification and simplification strategies. The following subsections describe how these outcomes differed by strategy and regimen type.

HbA1c

Both intensification and simplification strategies were associated with improved or maintained glycaemic outcomes (Figure 3). In intensification studies, HbA1c reductions were reported with insulin-dominant approaches, including newer-generation basal insulin formulations, high-concentration insulin, and bolus insulin intensification, as well as with add-on GLP-1RA, GIP/GLP-1RA, or SGLT2i therapy [24,25,26,27,28,29,30,31,33,35,36]. However, adding a DPP-4i or switching to a newer-generation basal insulin that compared with FRC, they did not improve glycaemic control [32,34].

Simplification studies generally reported reductions in or maintenance of HbA1c after switching from complex insulin regimens to FRCs, co-formulated insulin, basal insulin plus GLP-1RA, or GLP-1RA and/or SGLT2i therapy [34,38,39,40,41,42,43,44,45,46,47,48,49,50,51,52,53]. Across both strategy groups, several studies reported increased achievement of HbA1c targets, although target thresholds varied between 7% and 8% [27,29,30,37,39,51,54,55].

Body Weight

Body weight outcomes differed by strategy and regimen (Figure 3). Simplification studies generally reported body weight reductions after switching from complex insulin regimens to GLP-1RA-based therapy, GLP-1RA plus SGLT2i, or FRCs insulin [20,34,38,39,41,42,43,45,46,47,48,49,50,52,53]. Exceptions were reported among participants with previous GLP-1RA exposure and among those switched to high-concentration insulin [37,55].

In intensification studies, body weight outcomes were more variable. Insulin-dominant strategies were associated with weight gain [34,35,36]. Although one study observed weight gain with newer-generation basal insulin, the types did not differ significantly [33,34]. In contrast, intensification with GLP-1RA, GIP/GLP-1RA, or SGLT2i was associated with body weight reduction [24,25,26,27,28,30,31]. One BMI-stratified analysis found greater weight reduction in participants with BMI ≥ 30 versus <25 kg/m^2^ after adjustment for treatment and baseline characteristics. However, the effect of lixisenatide versus placebo on weight did not differ significantly across BMI categories [25]. Dose-related weight reduction was also reported with GIP/GLP-1RA therapy, with greater reductions at higher doses [27].

Insulin TDD

Changes in insulin TDD varied according to the type of modification strategy. Intensification studies more often reported increased TDD, particularly when insulin-dominant strategies or high-concentration insulin were used [33,34,36]. In contrast, reduced TDD was reported in intensification studies that incorporated GIP/GLP-1RA, GLP-1RA and/or SGLT2i therapy [27,29,30,31].

Most simplification studies reported reductions in TDD, although the extent of reduction varied across regimens. FRCs were most consistently associated with lower TDD in the majority of studies [34,47,49,50], but not for those previously on GLP-1RA [55]. IDegAsp showed mixed findings, with one study reporting no significant change and another larger study reporting a significant reduction [44,45]. High-concentration insulin was an exception, as it did not consistently reduce insulin requirements [37]. One subgroup analysis reported increased TDD among patients aged >65 years receiving FRC therapy [54].

Hypoglycaemia risk

Hypoglycaemia outcomes varied across simplification and intensification studies (Table 4 and Figure 3). Several simplification studies reported reduced hypoglycaemia or no significant difference relative to baseline or the comparator regimen [20,34,41,44,45,47,48,49,51,53,54], although increases occurred in some iGlarLixi prior-treatment subgroups [28,54]. In DUAL II Japan, descriptively lower rates with IDegLira versus degludec were observed among participants with BMI ≥ 25 to <30 kg/m^2^ or HbA1c < 9% [54].

In contrast, intensification studies reported mixed outcomes. Hypoglycaemia was more frequent with basal-bolus insulin than with basal insulin plus semaglutide or tirzepatide [24,28,35], and numerically more frequent with GLP-1RA than with SGLT2i therapy [29]. Differences versus the comparator were not significant for PEG-loxenatide or enavogliflozin [26,31]. Second-generation basal insulins showed low hypoglycaemia rates, with no significant difference between glargine U-300 and degludec U-100 [43].

Treatment burden

Treatment satisfaction and QOL outcomes were reported less frequently than glycaemic, weight, and insulin-dose outcomes (Figure 3). Simplification studies more often reported improved treatment satisfaction or QOL, particularly when injection frequency was reduced after switching from complex insulin regimens [39,40,41,44,47,50]. Evidence for QOL following intensification was limited; it came from a study of basal insulin plus GIP/GLP-1RA therapy [28].

Gastrointestinal adverse events were commonly reported in studies involving GLP-1RA or GIP/GLP-1RA therapy [25,27,28,29]. These events were reported less frequently in FRC studies or among participants with prior GLP-1RA exposure before simplification [52,55].

## 4. Discussion

This scoping review provides an integrated synthesis of ITM strategies among insulin-treated adults with T2DM and coexisting overweight or obesity. The included studies could be organised into four clinically relevant ITM domains: add-on therapy to insulin, switching to advanced insulin formulations, simplifying multiple daily injection (MDI) regimens with adjunctive therapy, and switching to FRCs. This framework illustrates how intensification and simplification can be complementary approaches rather than opposing pathways, addressing different clinical priorities, including glycaemic control, insulin exposure, treatment burden, body weight, and metabolic risk.

Intensification strategies were generally evaluated in populations with higher baseline HbA1c or greater treatment requirements, consistent with treatment escalation when glycaemic targets are not achieved [9,11]. However, conventional insulin intensification may increase regimen complexity, injection or titration burden, fear of hypoglycaemia, non-adherence, and clinician inertia [56]. In overweight or obese adults with T2DM, insulin-dominant intensification may also increase insulin TDD and body weight without proportional improvement in glycaemic outcomes [57]. In contrast, intensification incorporating GLP-1RA or SGLT2i was frequently associated with reduced insulin requirements and body weight, despite escalation of glucose-lowering treatment [9,11]. This distinction is clinically relevant because increasing insulin exposure may further exacerbate weight-related treatment concerns among patients with overweight and obesity. Consequently, concerns about weight gain may influence the choice of intensification strategy in individuals with higher baseline BMI. High insulin requirements also warrant consideration of whether the proposed regimen can meet glycaemic needs without unacceptable hypoglycaemia risk or treatment complexity. Rather than the designation of “intensification” itself, the therapeutic component used to intensify treatment may be more important in determining its reported metabolic and treatment-related effects.

Simplification strategies showed a comparatively consistent pattern, with lower insulin exposure, reduced injection burden, and body weight reduction commonly reported while glycaemic control was maintained or improved [19,58]. The predominance of GLP-1RA-based regimens and FRCs among these strategies is consistent with the increasing emphasis on weight management and reducing unnecessary treatment complexity in contemporary T2DM management [11,59]. Insulin therapy may therefore be viewed as a modifiable treatment platform rather than a fixed endpoint, particularly as newer insulin formulations and adjunctive glucose-lowering therapies become available [60,61]. Nevertheless, the applicability of these approaches may be influenced by medication access, affordability, and availability [4,59], and their feasibility may differ across healthcare settings [18,62]. Maintaining glycaemic control remains central when simplifying insulin treatment. Although simplification frequently reduced insulin TDD in the included studies, patients with high baseline insulin requirements require careful assessment before dose reduction. Progressive β-cell dysfunction further underscores the importance of considering residual insulin-secretory capacity when modifying treatment [14].

The different baseline profiles suggest that treatment choice should consider both metabolic needs and treatment burden. Higher HbA1c or insulin requirements may indicate a need for greater glucose-lowering potency, whereas hypoglycaemia risk and difficulty managing multiple injections may favour simplification [19]. Greater obesity may strengthen the rationale for weight-reducing adjunctive therapy, which can support glycaemic control while reducing insulin requirements. However, reducing insulin also requires consideration of residual β-cell function, and BMI alone cannot determine the preferred strategy [33,35,36,37]. Patient preferences and capacity to manage the regimen remain central to either approach [19].

Previous medication exposure may influence the effect of simplification strategies. Patients already receiving GLP-1RA therapy may experience less additional glycaemic or weight benefit after switching to an FRC, particularly if the GLP-1RA component of the FRC provides a lower effective dose than their previous regimen. Age and frailty may also require consideration when GLP-1RA- or SGLT2i-based approaches are introduced [63,64]. However, these characteristics were not consistently evaluated across the included studies and should not be interpreted as established predictors of treatment response or as criteria for selecting intensification or simplification strategies.

Taken together, the findings suggest that ITM outcomes are shaped less by the broad label of intensification or simplification than by the therapeutic components added, withdrawn, or replaced. Intensification incorporating weight-beneficial adjunctive therapies did not invariably increase insulin exposure or body weight, while simplification using FRCs or adjunctive non-insulin therapy did not appear to compromise glycaemic control. The four domains identified in this review may therefore help organise ITM evidence by regimen-modification pattern, although they should not be interpreted as a validated clinical decision algorithm.

Although this review focused on adults with overweight or obesity, most findings came from overall study populations rather than obesity-specific subgroup analyses. BMI-stratified analyses included those by Feng et al. [24] and Komatsu et al. [26]. Most findings describe outcomes in populations relevant to the review but do not establish whether obesity modifies the response to ITM. Both subgroup analyses reported glycaemic improvements across the BMI categories examined [24,26]. Feng et al. reported numerically higher proportions of lixisenatide-treated participants achieving glycaemic targets without weight gain in higher BMI categories [24]. However, these differences do not by themselves establish that the treatment effect relative to placebo varies by BMI. Komatsu et al. reported weight benefits with IDegLira versus degludec in insulin-experienced participants, without a significant treatment-by-BMI interaction for weight [26]. Evidence therefore remains insufficient to determine whether particular ITM strategies offer greater benefits in specific BMI categories. Most studies classified participants according to BMI, but few examined whether adiposity phenotype, insulin resistance, β-cell function, or body composition influenced treatment response. This evidence gap matters because BMI alone may underestimate metabolic risk, particularly in Asian populations, where higher body fat percentage and β-cell dysfunction may occur at lower BMI thresholds [18,65,66,67]. Consequently, patients with similar BMI may differ in their metabolic profile and response to insulin treatment modification. Variability in response to glucose-lowering therapy has also been associated with clinical and genetic factors, although their utility for treatment selection remains uncertain [68,69]. Although therapies that target both glycaemic control and obesity-related metabolic dysfunction may be particularly relevant in this population, current evidence remains insufficient to determine which obesity phenotypes are most likely to benefit from specific ITM strategies [70,71].

Regional generalisability also remains an important consideration. Much of the evidence was generated in Western or relatively well-resourced settings, whereas evidence from Asian and lower-resource settings was more limited. Differences in metabolic characteristics, medication access, affordability, insulin availability, and healthcare infrastructure may affect the feasibility and sustainability of individual ITM strategies [18,56]. Emerging Asian evidence demonstrates the use of newer GLP-1RA- and SGLT2i-based therapies [72,73], but direct comparative evidence examining their role within ITM remains limited. Successful implementation may also depend on adequate insulin titration, self-monitoring, patient education, and follow-up [8]. More broadly, treatment costs, access to newer therapies, and healthcare system resources may influence the translation of ITM strategies into routine practice [74,75]. These contextual factors were not consistently evaluated in the included studies and warrant more systematic investigation.

This review has several limitations. Only English-language publications were included, which may have reduced the representation of evidence from some regions. The interventions were classified as intensification or simplification using the review’s operational definitions, which did not necessarily correspond to the terminology used in the original studies. These differences limit direct comparisons because reported outcomes may reflect baseline glycaemic status, patient selection or study methods as well as the treatment strategy. Differences in sample size affect the precision of estimates, while differences in follow-up duration affect the assessment of sustained benefits and adverse events. Accordingly, the observed outcome patterns should be interpreted descriptively and do not establish the relative effectiveness or safety of intensification and simplification. The available evidence was further limited by the restriction to publications from 2021 to March 2026. This window focused the review on contemporary insulin treatment modification strategies but may have excluded earlier clinically relevant evidence. Moreover, this review was limited by a small number of studies directly comparing intensification and simplification strategies, and by the uncommon use of obesity-specific subgroup analyses. Consequently, the findings should be interpreted as a mapping of recent evidence rather than a comprehensive historical synthesis of all insulin treatment modification strategies. As this was a scoping review, the objective was to map available evidence rather than compare treatment effectiveness; therefore, formal risk-of-bias assessment was not undertaken.

## 5. Conclusions

In conclusion, insulin treatment modification in adults with T2DM and overweight or obesity should be individualised rather than restricted to a single pathway. Relevant considerations include baseline glycaemic status, insulin regimen complexity, insulin requirement, adiposity-related risk, prior medication exposure, and safety. Although this scoping review cannot establish the superiority of either strategy, simplification may be considered for patients with long-standing, moderately uncontrolled T2DM receiving complex insulin regimens. This approach may be particularly relevant when reducing body weight, insulin exposure, or injection burden is clinically important. Intensification remains relevant for patients with suboptimal glycaemic control associated with inadequate insulin dosing or insufficient treatment potency, although it may increase treatment burden. Newer insulin co-formulations, FRCs, and replacement of prandial insulin with non-insulin agents that offer weight benefits may provide useful treatment modification options. Further comparative studies are needed to establish their relative effectiveness and safety across different obesity phenotypes, insulin requirements, and healthcare settings.

## Figures and Tables

**Figure 1:**
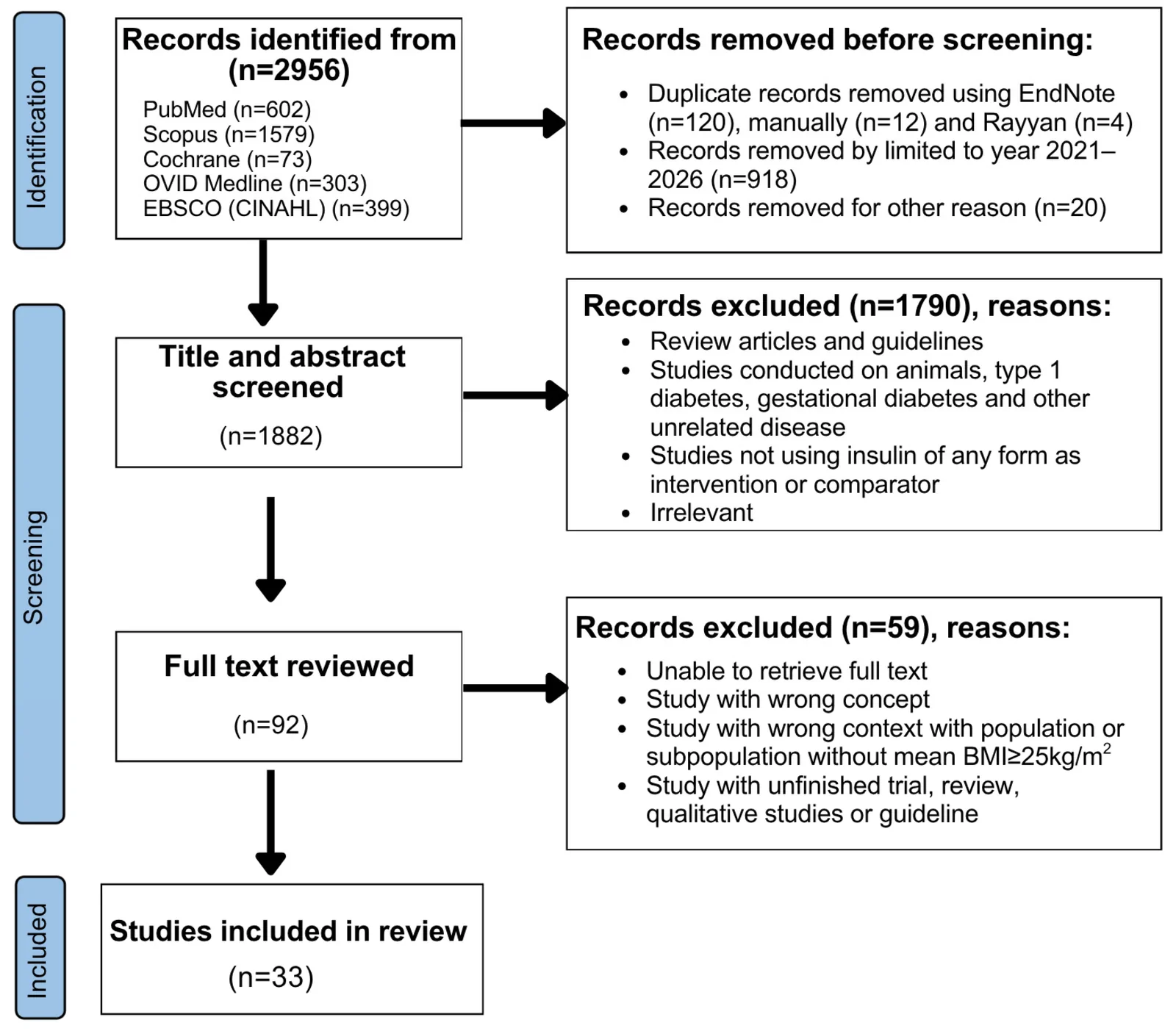
PRISMA-Scr Flow Diagram of Study Selection Progress.

**Figure 2:**
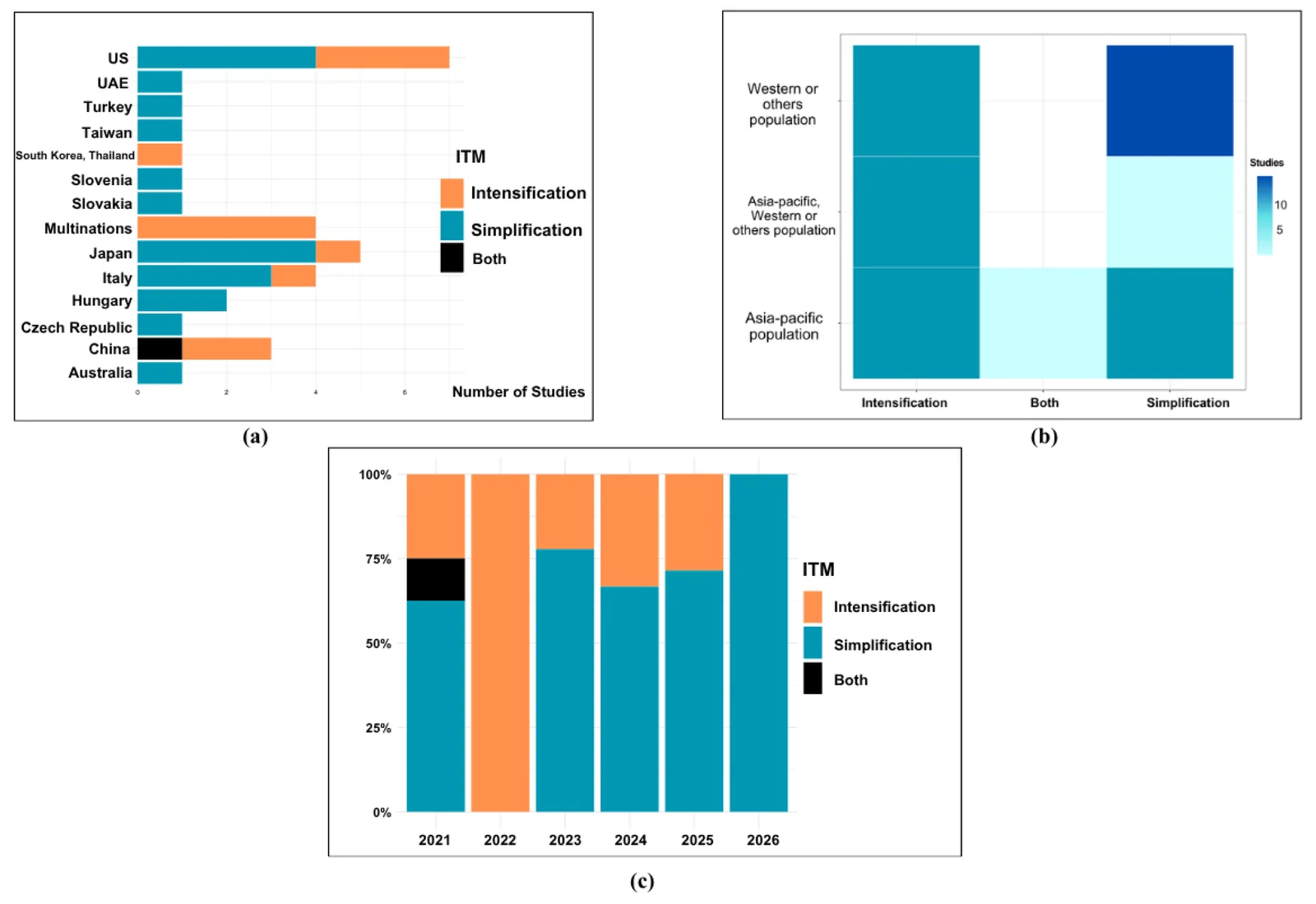
(**a**) Distribution of Studies by Country. US, United States; UAE, United Arab Emirates. (**b**) Distribution of Studies by Population Region and ITM Category. Darker colours indicate a greater number of studies. (**c**) Percentage of Studies by ITM Category and Publication Year, from 2021 to March 2026. The study contributing to both treatment categories is represented as “Both” and counted once.

**Figure 3:**
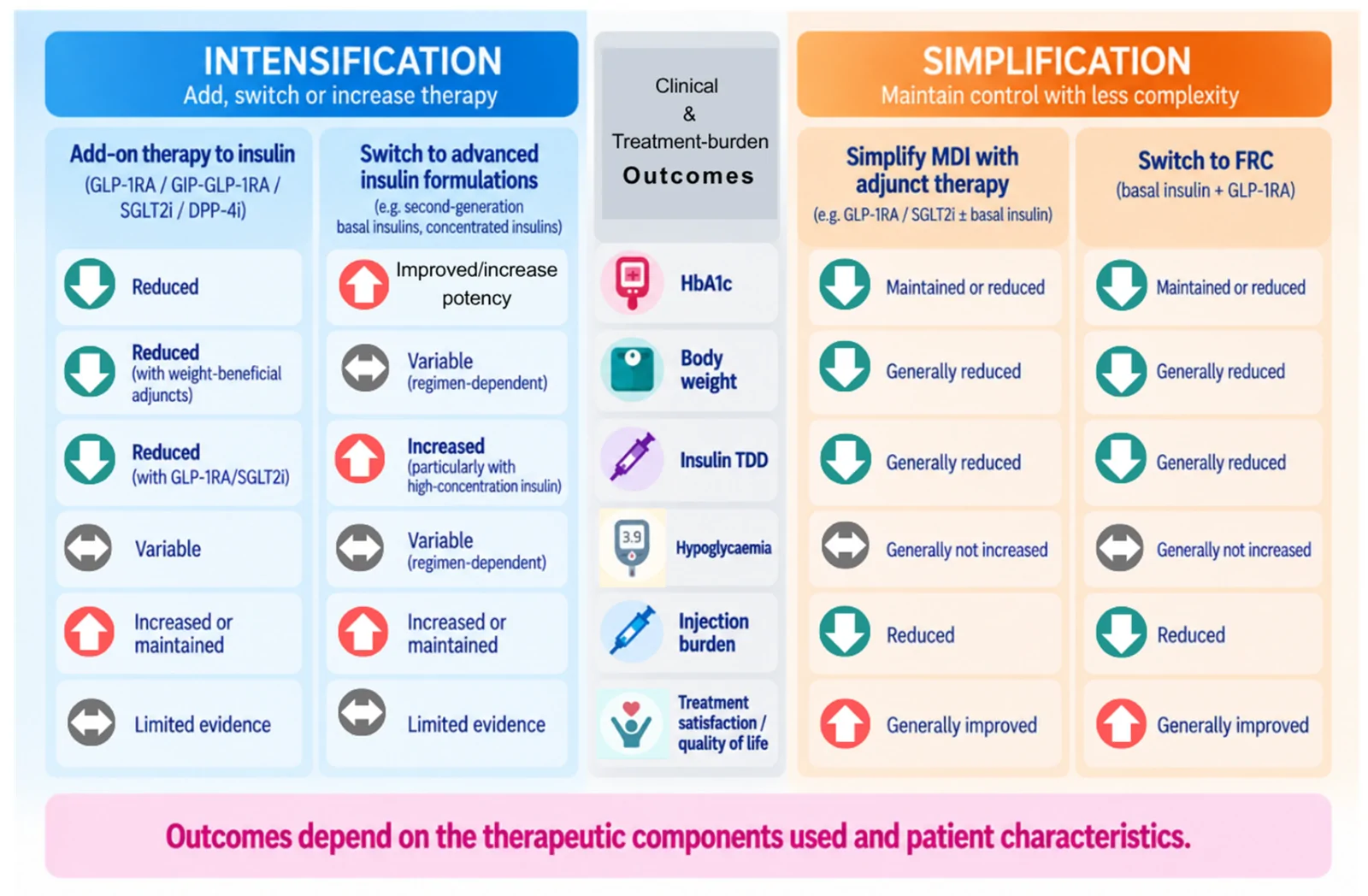
Summary of Significant Outcomes Comparison.

**Table 1:** Inclusion Criteria for This Scoping Review.

PCC	Description
Population	Adults aged ≥ 18 years with T2DM and overweight or obese (BMI ≥ 25 kg/m^2^) who were receiving insulin before treatment modification
Concept	ITM strategies including intensification or simplification, used among T2DM patients
Context	Current insights and evidence-based practice related to ITM in global studies of overweight or obese T2DM patients.
Types of sources	Primary and secondary quantitative studies, including post hoc analyses

**Table 2:** Operational Definitions.

**Insulin** **Intensification**	Escalation of glucose-lowering treatment by adding therapy or modifying the existing insulin regimen to enhance glucose-lowering capacity, irrespective of changes in total daily insulin dose.
**Insulin** **Simplification**	Modification of an existing insulin regimen to reduce injection frequency, the number of separately administered treatment components, or administration complexity relative to the previous regimen, irrespective of changes in total daily insulin dose.

Changes in insulin dose were reported as an outcome of ITM strategies. Intensification and simplification are not mutually exclusive. A treatment modification may meet both definitions when glucose-lowering therapy is escalated while the insulin regimen is simplified.

**Table 3:** Characteristics of Included Studies by Treatment Approach.

Baseline Characteristics	Intensification		Simplification	
	Value (*n*)	Percentage %	Value (*n*)	Percentage %
**Age Range**	53.9–70.3 years old	-	51.8–70.2 years old	-
**Duration of T2DM** **Diagnosed Range**	10.4–20 years	-	7.8–19 years	-
**Baseline HbA1c Range**	7.57–9.3%	-	6.4–9.6%	-
**Classification of Baseline BMI**				
Overweight BMI ≥ 25 to <30 kg/m^2^				
	5	38.5%	6	28.6%
Obesity BMI ≥ 30 kg/m^2^				
	8	61.5%	15	71.4%
**Baseline Insulin TDD Range**				
TDD < 100 u/day	24.5–73.3 u/day	-	8.9–70 u/day	-
TDD ≥ 100 u/day	183–336 u/day	-	107–188 u/day	-
**Study** **Characteristics**				
**Study Design**				
Interventional Studies	6	46.2%	8	38.1%
Observational Studies	4	30.8%	11	52.4%
Post hoc Analysis	3	23.1%	2	9.5%
**Sample Size**				
0–100	2	15.4%	7	33.3%
101–500	6	46.2%	12	57.1%
>500	5	38.5%	2	9.5%
**Follow-up Duration**				
12–16 weeks/3–4 months	1	7.7%	2	9.5%
24–30 weeks/5.5–7.5 months	9	69.2%	12	57.1%
36–52 weeks/9–12 months	3	23.1%	2	9.5%
>12 months	0	0%	5	23.8%

Data were presented as the number of studies by relevant treatment category, value (*n*), percentage (%), and the range of baseline and study characteristics. Abbreviations: HbA1c, glycated haemoglobin; BMI, Body Mass Index, TDD, Total Daily Dose; u, Units.

**Table 4:** Summary and Key Findings of Insulin Intensification and Simplification Strategies.

Baseline Characteristics	Overweight/ Obesity	Insulin Regimen	Summary of Findings	Author (Year)
Intensification				
aged 58 years, BMI 31.7 kg/m^2^, duration 13 years, HbA1c 8.2%, TDD 43–46 u/day on insulin basal and metformin	Obesity	Basal and GLP-1RA (semaglutide 1.0 mg OW)	HbA1c ↓ BW ↓ HYPO ↓ [compared to BB]	Lingvay et al. (2023) ** [24]
aged 53.9 years, BMI 26.8 kg/m^2^, duration 10.4 years, HbA1c 8%, Asian, TDD 37.8–51 u/day, on Insulin glargine ± metformin	Overweight	Basal and GLP-1RA (Lixisenatide)	HbA1c ↓ BW ↓ [compared to placebo]	Feng et al. (2021) ** [25]
aged 58 years, BMI 26.8 kg/m^2^, duration 11 years, HbA1c 8.55%, on basal insulin	Overweight	Basal and GLP-1RA (PEG-Loxe)	HbA1c ↓ BW ↓ HYPO: 10.4% versus 6.3% [compared to basal insulin]	Liu et al. (2025) [26]
aged 61 years, BMI 33.4 kg/m^2^, duration 13.3 years, HbA1c 8.31%, TDD 32–35 u or >0.25 u/kg/day on insulin basal	Obesity	Basal and GIP/GLP-1RA (Tirzepatide 5, 10, 15 mg)	HbA1c ↓ BW ↓ TDD ↓ HYPO: 14.2–19.3% versus 12.5% [compared to placebo]	Dahl et al. (2022) [27]
aged 58.6 years, BMI 33.2 kg/m^2^, duration 13.7 years, HbA1c 8.8%, TDD 46 u/day on insulin basal ± metformin/OGLDs	Obesity	Basal and GIP/GLP-1RA (tirzepatide 5 mg, 10 mg, 15 mg OW)	HbA1c ↓ BW↓ HYPO: 10.6% versus 48% QOL: improved [compared to BB]	Boye et al. (2024) [28]
aged 60 years, BMI 37 kg/m^2^, HbA1c 9.3%/9.5%, TDD 72.2 u/day, on insulin basal ± bolus ± OGLDs	Obesity	Basal/BB and GLP-1RA (liraglutide, dulaglutide, exenatide)	HbA1c ↓ TDD ↓ BW↓ HYPO: 21% versus 11% with SGLT2i	George et al. (2022) [29]
aged 60 years, BMI 37 kg/m^2^, HbA1c 9.3%/9.5%, TDD 73.3 u/day, on insulin basal ± bolus ± OGLDs	Obesity	Basal/BB and SGLT2i (empagliflozin, canagliflozin)	HbA1c ↓ TDD: non-significant increase BW: no significant difference HYPO: 11% versus 21% with GLP-1RA	
aged 69 years, BMI 40.8 kg/m^2^, duration 20 years, HbA1c 8.9%, TDD 183 u/day, on various insulin regimen, mainly BB	Obesity	BB and GLP-1RA (semaglutide)	HbA1c ↓ BW ↓ TDD ↓	Meyer et al. (2023) [30]
aged 61 years, BMI 26.4 kg/m^2^, duration 15.1–16.1 years, HbA1c 8.6%, C-peptide 0.29–0.35 nmol/L, on insulin basal/premixed	Overweight	Basal/Premixed and SGLT2i (Enavogliflozin 0.3 mg/day)	HbA1c ↓ BW ↓ TDD ↓ HYPO: 29.2%, no significant difference [compared to placebo]	Hong et al. (2025) [31]
aged 57.1 years, BMI 26.6 kg/m^2^, duration 13 years, HbA1c 7.57%, on MDI BB + 750 mg/day metformin	Overweight	BB and DPP-4i (Vildagliptin 100 mg/day)	No significant differences in HbA1c, BW, TDD and HYPO [compared to high-dose metformin]	Yoshikawa et al. (2022) [32]
aged 69.9–70.3 years, BMI 30.51–30.3 kg/m^2^, duration 18.7–19 years, HbA1c 8.7%, TDD 57–58 u/day, on insulin basal	Obesity	Gen 2 Basal (insulin Glargine 300/insulin Degludec 100)	HbA1c ↓ BW: no significant difference TDD ↑ HYPO: 1.09–1.57 event/person/month, no significant difference between Glargine and Degludec	Buzzetti et al. (2022) [33]
aged 54.7 years, BMI 27.4 kg/m^2^, duration 11.4 years, HbA1c 8.94%, TDD 24.5–25.3 u/day, basal insulin + metformin (≥1500 mg/day)	Overweight	Insulin Degludec	HbA1c ↑ BW ↑ TDD ↑ HYPO: 14.6%, significantly higher [compared to IDegLira]	Pei et al. (2021) # [34]
aged 61.2 years, BMI 31.5 kg/m^2^, duration 13.4 years, HbA1c 8.6%, TDD 40.9 u/day on insulin basal, metformin ± 1 OGLDs	Obesity	Basal and Insulin Aspart (thrice daily)	HbA1c ↓ BW ↑ HYPO: 60.4% versus 37.8%, significantly higher [compared to basal+ GLP-1RA]	Kellerer et al. (2022) [35]
aged 61.2 years, BMI 31.5 kg/m^2^, duration 13.4 years, HbA1c 8.6%, TDD 40.2 u/day on insulin basal, metformin ± 1 OGLDs	Obesity	Basal and GLP-1RA (semaglutide)	HbA1c ↓ BW ↓ HYPO: 37.8% versus 60.4%, significantly lower [Compared to BB]	
aged 57.1 years, BMI 41.1–39.1 kg/m^2^, duration 17.1 years, HbA1c 8.56–8.84%, TDD 271–336 u/day on MDI high insulin dose	Obesity	U-500 Insulin (CSII)	HbA1c ↓ TDD: no significant difference BW ↑ HYPO: no significant difference [compared to U-500 MDI]	Hood et al. (2021) ** [36]
**Simplification**				
aged 63.9 years, BMI 39.7 kg/m^2^, HbA1c 9.5%, TDD 188.2 u, on MDI insulin > 1 type insulin	Obesity	U-500 Insulin (twice/thrice daily)	HbA1c ↓ BW ↑ TDD ↑ HYPO: 71.9%, significantly increased from baseline	Chen et al. (2021) [37]
aged 61.7 years, BMI 32.4 kg/m^2^, duration 11.5 years, HbA1c 8.2%, on basal/BB ± OGLDs	Obesity	Basal and GLP-1RA (semaglutide 0.5 mg–1.0 mg)	HbA1c ↓ BW ↓ TDD ↓	Bonora et al. (2025) [38]
aged 68.6 years, BMI 35.5 kg/m^2^, duration 18.5 years, HbA1c 6.8%, TDD 70 u/day on insulin BB	Obesity	Basal and GLP-1RA (semaglutide 1.0 mg)	HbA1c ↓ BW ↓ TDD ↓ HYPO: no significant difference Satisfaction score: higher [compared to MDI insulin]	Rodriguez et al. (2025) [39]
aged 57.1 years, BMI 26.8/27 kg/m^2^, duration 7.8/10.4 years, HbA1c 6.7%, TDD 29.6–34.5 u/day on BB	Overweight	Basal and GLP-1RA/SGLT2i (liraglutide, canagliflozin)	HbA1c: no significant difference BW ↓ TDD ↓ Severe HYPO: 0%, from baseline INJ frequency↓ QOL score ↑	Ando et al. (2021) [40]
aged 51.8 years, BMI 36.27 kg/m^2^, duration 15.75 years, HbA1c 9.69%, TDD 107 u/day, on complex insulin ≥ 2 injections	Obesity	Basal and SGLT2i + GLP-1RA (Empagliflozin + Dulaglutide)	HbA1c ↓ BW: no significant difference TDD ↓ HYPO: 8.3% versus 30% Satisfaction score: higher [compared to MDI insulin]	Naing et al. (2021) [41]
aged 58 years, BMI 27.7 kg/m^2^, duration 12 years, HbA1c 8.6%, TDD 61 u/day, on premixed insulin ± metformin	Overweight	Basal and GLP-1RA (liraglutide)	HbA1c ↓ BW ↓ TDD ↓	Liu et al. (2023) [42]
aged 61.3 years, BMI 35.5 kg/m^2^, duration 13.1 years, HbA1c 8.6%, TDD 28.6 u/day on insulin BB	Obesity	Basal and GLP-1RA (Liraglutide, Dulaglutide, Exenatide, Lixisenatide, IDegLira, iGlarLixi)	HbA1c ↓ BW ↓ TDD ↓ [compared to BB + GLP-1RA]	Bonora et al. (2021) [43]
aged 64.8 years, BMI 30.13 kg/m^2^, duration 17.3 years, HbA1c 9%, TDD 38.2 u/day on insulin basal/BB/premixed	Obesity	IDegAsp (once/twice daily)	HbA1c ↓ BW: no significant difference TDD ↓ HYPO: 0.82 events/month, significantly reduced from baseline INJ frequency ↓	Semiz et al. (2023) [44]
aged 67.2 years, BMI 31.6 kg/m^2^, duration 19 years, HbA1c 9.1%, TDD 56.3 u/day on various insulin regimen	Obesity	IDegAsp (once/twice daily)	HbA1c ↓ BW↓ TDD: no significant difference HYPO: 10.9%, non-significant reduction from baseline	Fulcher et al. (2024) [45]
aged 63.8 years old, BMI 33.01 kg/m^2^, duration 9.7 years, HbA1c 6.4%, C-peptide 3.96 ng/mL, TDD 43.2 u/day, on insulin BB/premixed	Obesity	IDegLira (once daily)	HbA1c: no significant difference BW↓ TDD↓ HYPO: 16.7%, reduced from baseline	Taybani et al. (2023) [46]
aged 64.9 years, BMI 33.9 kg/m^2^, duration 16.1 years, HbA1c 8.6%, C-peptide levels 0.75 ± 0.43 nmol/L, TDD 55.6 u/day on insulin BB	Obesity	IDegLira (once daily)	HbA1c ↓ BW↓ TDD↓ HYPO: 4.7%, significantly reduced from baseline Treatment satisfaction: 69% of participants very satisfied	Martinka et al. (2023) [47]
aged 67.2 years, BMI 30.3 kg/m^2^, duration 15.7 years, HbA1c 8.5%, TDD 43.7 u/day on BB ± OGLDs	Obesity	IDegLira (once daily)	HbA1c ↓ BW ↓ HYPO: reduction from baseline	Fadini et al. (2024) [48]
aged 67.2 years, BMI 30.3 kg/m^2^, duration 15.7 years, HbA1c 8.5%, TDD 16.9 u/day on basal ± OGLDs	Obesity	IDegLira (once daily)	HbA1c ↓ BW ↓ HYPO: reduction from baseline	
aged 70.2 years, BMI 31 kg/m^2^, duration 15.2 years, HbA1c 7.39%, TDD 44.5 u/day, on premixed insulin	Obesity	IDegLira (once daily)	HbA1c ↓ BW ↓ TDD ↓ HYPO: no significant difference [compared to premixed insulin]	Kravos Tramsek et al. (2025) [49]
aged 63 years, BMI 30.27 kg/m^2^, duration 11 years, HbA1c 8.25%, c-peptide 4.05 ng/mL, TDD 48 u/day, on insulin BB + OGLDs	Obesity	iGlarLixi or IDegLira (once daily)	HbA1c ↓ BW ↓ HYPO ARR: 27.35%, significantly lower [compared to conservative insulin treatment]	Fejes et al. (2025) [20]
age 66.2 years, BMI 33.6 kg/m^2^, duration of 17.5 years, HbA1c 7.9% (non-compliance/fasting C-peptide levels above 0.37 nmol/L) TDD 63 u, on insulin BB ± metformin/SGLT2i	Obesity	iGlarLixi (once daily)	HbA1c: no significant difference BW ↓ TDD ↓ HYPO: 5.5%, significantly lower Satisfaction score: higher [compared to MDI insulin]	Novodvorský et al. (2026) [50]
aged 58.9 years, BMI 35.9 kg/m^2^, duration 13.3 years, HbA1c 9.6%, on BB	Obesity	iGlarLixi (once daily)	HbA1c ↓ BW: no significant difference HYPO: reduced from baseline	Giorgino et al. (2025) [51]
aged 61.6, BMI 26.8 kg/m^2^, HbA1c 8.96%, on insulin basal/BB/premixed	Overweight	iGlarLixi (once daily)	HbA1c ↓ BW ↓ HYPO: 14.4%, increased from baseline	Matsuhisa et al. (2023) [52]
aged 51.9 years, BMI 30.5 kg/m^2^, duration 12.2 years, HbA1c 9.29%, TDD 30.4 u/day, on insulin basal ± OGLDs	Obesity	IDegLira (once daily)	HbA1c ↓ BW ↓ TDD ↑ HYPO: reduced from baseline	Abusnana et al. (2023) [53]
aged 54.7 years, BMI 27.4 kg/m^2^, duration 11.46 years, HbA1c 8.94%, TDD 25.1 u/day, basal insulin + metformin (≥1500 mg/day)	Overweight	IDegLira (once daily)	HbA1c ↓ BW ↓ TDD ↓ HYPO: 11.3%, significantly lower [compared to insulin degludec]	Pei et al. (2021) # [34]
aged <65 years, HbA1c ≥ 8.0 < 9.0%, BMI ≥ 25 to <30 kg/m^2^, on insulin basal/premixed (from majority of subgroups)	Overweight	IDegLira (once daily)	HbA1c ↓ BW↓ TDD ↓ HYPO: no significant difference [compared to insulin degludec]	Komatsu et al. (2021) ** [54]
aged 58.5–63.2 years, BMI 26.2 to 27.2 kg/m^2^, duration ≥ 10 years, HbA1c 8.49–9.18%, TDD 8.9–12.2 u/day, on various insulin regimens; basal/premixed/BB ± OGLDs	Overweight	iGlarLixi (once daily)	HbA1c ↓ BW ↓ TDD: no significant difference HYPO: 10.8–28.2%, increased from baseline	Miyoshi et al. (2023) ** [55]
aged 58.5–63.2 years, BMI 26.2 to 27.2 kg/m^2^, duration ≥ 10 years, HbA1c 8.49–9.18%, TDD 8.9–12.2 u/day, on insulin basal + GLP-1RA ± OGLDs	Overweight	iGlarLixi (once daily)	HbA1c: no significant difference BW: no significant difference TDD ↑ HYPO: 15.4%, increased from baseline	

Abbreviations: ARR, Absolute Risk Reduction; BB, Basal Bolus; BW, Body Weight; CSII, Continuous Subcutaneous Insulin Infusion; Duration, Duration of T2DM Diagnosed; GEN 2, Generation 2; HYPO, Hypoglycaemia Events; INJ, Injection; OGLDs, Oral Glucose Lowering Drugs; PEG-Loxe, Polyethylene Glycol Loxenatide; QOL, Quality of Life; u, Units; U-500, Insulin Regular Concentrated Insulin. Symbols: ↓ significantly reduce; ↑ significantly increase; # Two distinct treatment strategy; ** Post-hoc Subgroups Analysis: Hypoglycaemia outcomes are reported as the percentage of participants experiencing events, event rates with the stated units, or absolute risk reductions, as specified in each entry. Event definitions and observation periods follow the original studies. Outcome comparisons refer to baseline or the indicated treatment group.

**Table 5:** Four Types of Insulin Treatment Modification Strategies.

Domain	Strategy	Description
Intensification Strategies	[A]. Add-on therapy to insulin	Adding bolus insulin or non-insulin glucose-lowering agents, such as GLP-1RA/SGLT2i/DPP-4i, to an existing insulin regimen to improve glycaemic control may result in more injections and/or a higher number of medications
	[B]. Switch to advanced insulin formulations	Switching to a newer-generation basal analogue or to more concentrated insulin pump therapy when the change constitutes treatment escalation relative to the previous regimen
Simplification Strategies	[C]. Simplify complex insulin regimen with adjunct therapy	Simplify a complex insulin regimen by replacing prandial or premixed insulin with non-insulin glucose-lowering agents such as GLP-1RA/SGLT2i/DPP-4i, while retaining basal insulin, prioritising insulin regimen simplification.
	[D]. Switch to FRCs	Switching of various insulin regimen to a simpler, single-injectable agent for FRCs, such as IDegAsp/IDegLira/iGlarLixi, when this reduces injection frequency, separate administration of treatment components, or overall administration complexity relative to the previous regimen.

Complex insulin regimen is an insulin regimen with more than one type or number of injections.

## Data Availability

The original contributions presented in this study are included in the article/Supplementary Material. Further inquiries can be directed to the corresponding author.

## Supplementary Materials

The following supporting information can be downloaded at: https://www.mdpi.com/article/10.3390/ijms27209050/s1.

## Abbreviations

The following abbreviations are used in this manuscript:

BMI	Body Mass Index
DPP-4i	Dipeptidyl Peptidase-4 Inhibitors
FRC	Fixed-ratio combination
GIP/GLP-1RA	Dual Glucose-Dependent Insulinotropic Polypeptide and Glucagon-Like Peptide 1 Receptor Agonists
GLP-1RA	Glucagon-Like Peptide-1 Receptor Agonists
HbA1c	Glycated haemoglobin
IDegAsp	insulin Degludec and insulin Aspart
IDegLira	insulin degludec and liraglutide
iGlarLixi	insulin glargine and lixisenatide
ITM	Insulin treatment modification
MDI	Multiple daily injection
SGLT2i	Sodium-Glucose Cotransporter-2 Inhibitors
T2DM	Type 2 Diabetes Mellitus
TDD	Total Daily Dose
