# Supplementary material for: Insulin Intensification and Simplification Strategies in Patients with Type 2 Diabetes and Overweight or Obesity: A Scoping Review
Source: Int J Mol Sci. 2026 Oct 12;27(20):9050. doi: 10.3390/ijms27209050 (PMC13704631; doi:10.3390/ijms27209050)
Supplement: Supplementary file 1 [file ijms-27-09050-s001.zip › ijms-4587323-supplementary.pdf]

Supplementary Material

Insulin Intensification and Simplification Strategies in Patients with Type 2 Diabetes and Overweight or Obesity: A Scoping Review

Table of Contents

Supplementary Table S1: Search details for PubMed 2

Supplementary Table S2: Search details for Scopus 2

Supplementary Table S3: Search details for Cochrane 3

Supplementary Table S4: Search details for OVID Medline 4

Supplementary Table S5: Search details for EBSCO CINAHL 4

Supplementary Table S1: Search details for PubMed

Date of Search: 23/2/2026

Search	Query	Results
#5	Search: #1 AND #2 AND #3 AND #4	**602**
#4	Search: ((((((((((((((((Obesity[MeSH Terms]) OR (Diabesity[MeSH Terms])) OR (Overweight[MeSH Terms])) OR (Adiposity[MeSH Terms])) OR (Body mass index[MeSH Terms])) OR (Diabetes-related obesity[Title/Abstract])) OR (Diabetic obesity[Title/Abstract])) OR (Obesity-induced diabetes[Title/Abstract])) OR (Obes[Title/Abstract])) OR (Overweight[Title/Abstract])) OR (Adiposity[Title/Abstract])) OR (Body mass index[Title/Abstract])) OR (BMI[Title/Abstract])) OR (Body fat percentage[Title/Abstract])) OR (Waist circumference[Title/Abstract])) OR (Waist hip ratio[Title/Abstract])) OR (Visceral fat[Title/Abstract])) OR (Metabolic obesity[Title/Abstract])	678,312
#3	Search: (((((((((((((((Diabetes Mellitus, Type 2[MeSH Terms]) OR (Diabetes Mellitus, Type 2/drug therapy[MeSH Terms])) OR (Diabetes Mellitus, Noninsulin Dependent[Title/Abstract])) OR (Diabetes Mellitus, Adult Onset[Title/Abstract])) OR (NIDDM[Title/Abstract])) OR (Type 2 Diabetes Mellitus[Title/Abstract])) OR (adult-onset diabetes[Title/Abstract])) OR (type 2 diabet*[Title/Abstract])) OR (high blood sugar[Title/Abstract])) OR (Dysglycemia[Title/Abstract])) OR (Hyperglycemia[Title/Abstract])) OR (High blood glucose[Title/Abstract])) OR (Type II Diabetes[Title/Abstract])) OR (Type II Diabetes Mellitus[Title/Abstract])) OR (T2DM[Title/Abstract])) OR (Non-insulin dependent diabetes[Title/Abstract])) OR (Noninsulin dependent diabetes mellitus[Title/Abstract])	334853
#2	Search: (((((((((((insulin intensification[Title/Abstract]) OR (de-intensification[Title/Abstract])) OR (insulin titration[Title/Abstract])) OR (insulin dose adjustment[Title/Abstract])) OR (step up therapy[Title/Abstract])) OR (simplification[Title/Abstract])) OR (Step down[Title/Abstract])) OR (deintensification[Title/Abstract])) OR (modification[Title/Abstract])) OR (clinical inertia[Title/Abstract])) OR (de-prescribing[Title/Abstract])) OR (optimisation[Title/Abstract])	433,896
#1	Search: ((((((((((((Insulin[MeSH Terms]) OR (Insulin/administration and dosage[MeSH Terms])) OR (Insulin, Regular, Human[MeSH Terms])) OR (Insulin/therapeutic use[MeSH Terms])) OR (insulin*[Title/Abstract])) OR (Basal Insulin[Title/Abstract])) OR (Bolus Insulin[Title/Abstract])) OR (premixed insulin[Title/Abstract])) OR (insulin analog*[Title/Abstract])) OR (insulin dosage[Title/Abstract])) OR (insulin regimen*[Title/Abstract])) OR (insulin therapy[Title/Abstract])) OR (basal-bolus[Title/Abstract])	493,254

Supplementary Table S2: Search details for Scopus

Date of search: 24 Feb 2026

Search	Query	Results
	Search: #1 AND #2 AND #3 AND #4 with<br>Limit to,<br>1. Subject area: medicine, pharmacology, toxicology and pharmaceutics<br>2. Years: 2021-2026<br>3. Document type: Article<br>4. Language: English	**1579**
#5	Search: #1 AND #2 AND #3 AND #4	10368
#4	((TITLE-ABS-KEY (Obesity) OR TITLE-ABS-KEY (overweight obesity) OR TITLE-ABS-KEY (adiposity) OR TITLE-ABS-KEY (Body mass INDEX) OR TITLE-ABS-KEY (bmi) OR TITLE-ABS-KEY (body weight) OR TITLE-ABS-KEY (overweight)))	1,804,455

#3	((TITLE-ABS-KEY (intensification) OR TITLE-ABS-KEY (de intensification) OR TITLE-ABS-KEY (dose titration) OR TITLE-ABS-KEY (step down) OR TITLE-ABS-KEY (simplification) OR TITLE-ABS-KEY (optimisat*) OR TITLE-ABS-KEY (insulin step up) OR TITLE-ABS-KEY (deintensification) OR TITLE-ABS-KEY (insulin modification) OR TITLE-ABS-KEY (increas* dose) OR TITLE-ABS-KEY (decreas* dose) OR TITLE-ABS-KEY (deprescrib*) OR TITLE-ABS-KEY (intensifi*) OR TITLE-ABS-KEY (deintensifi*) OR TITLE-ABS-KEY (step up therapy) OR TITLE-ABS-KEY (drug dose titration) OR TITLE-ABS-KEY (dose adjustment) OR TITLE-ABS-KEY (total daily dose)))	2070678
#2	((TITLE-ABS-KEY (insulin) OR TITLE-ABS-KEY (basal insulin) OR TITLE-ABS-KEY (bolus insulin) OR TITLE-ABS-KEY (premixed insulin) OR TITLE-ABS-KEY (analog W/0 insulin) OR TITLE-ABS-KEY (insulin regimen) OR TITLE-ABS-KEY (insulin treatment) OR TITLE-ABS-KEY (co formulation W/0 insulin) OR TITLE-ABS-KEY (insulin treatment) OR TITLE-ABS-KEY (insulin therapy) OR TITLE-ABS-KEY (insulin use) OR TITLE-ABS-KEY (basal bolus insulin) OR TITLE-ABS-KEY (basal bolus therapy) OR TITLE-ABS-KEY (insulin W/0 administration) OR TITLE-ABS-KEY (insulin W/0 therapeutic) OR TITLE-ABS-KEY (insulin , regular , human)))	964,336
#1	((TITLE-ABS-KEY (Diabetes Mellitus , Noninsulin Dependent) OR TITLE-ABS-KEY (Diabetes Mellitus , Adult Onset) OR TITLE-ABS-KEY (niddm) OR TITLE-ABS-KEY (Type 2 Diabetes Mellitus) OR TITLE-ABS-KEY (adult-onset diabetes) OR TITLE-ABS-KEY (type 2 diabet*) OR TITLE-ABS-KEY (Type II Diabetes Mellitus) OR TITLE-ABS-KEY (Type II Diabetes) OR TITLE-ABS-KEY (t2dm) OR TITLE-ABS-KEY (Non insulin dependent diabetes) OR TITLE-ABS-KEY (Noninsulin dependent diabetes mellitus) AND NOT TITLE-ABS-KEY (diabetes mellitus type 1) AND NOT TITLE-ABS-KEY (diabetes , gestational)))	296,979

Supplementary Table S3: Search details for Cochrane

Date of search: 25 Feb 2026

Search	Query	Results
#1	(insulin):ti,ab,kw (Word variations have been searched)	81317
#2	treatment modification	12834
#3	MeSH descriptor: [Insulins] explode all trees	18669
#4	MeSH descriptor: [Obesity] explode all trees	22903
#5	insulin intensificati*	440
#6	insulin deintensificati*	15
#7	insulin daily dose	6298
#8	MeSH descriptor: [Diabetes Mellitus, Type 2] explode all trees	27496
#9	insulin optimization	476
#10	(#1 OR #3) AND (#2 OR #5 OR #6 OR #9) AND #8	1255
#11	MeSH descriptor: [Body Mass Index] explode all trees	14264
#13	titration	13850
#14	(#1 OR #3) AND (#5 OR #6 OR #9 OR #13 OR #7) AND #8 AND (#4 OR #11)	271
#15	MeSH descriptor: [Body Weight] explode all trees	41926
#16	(#1 OR #3) AND (#5 OR #6 OR #9 OR #13 OR #7) AND #8 AND (#4 OR #11 OR #15)	558
	Cochrane review	4
	Trials	554
	Search limit to,<br>Publication from years 2021 to 2026	review:1<br>Trials:**73**

Supplementary Table S4: Search details for OVID Medline

Date of search: 23 Feb 2026

Search	Query	Results
#5	Search: #1 AND #2 AND #3 AND #4 without filters	**303**
#4	Search: (obesity or obesity* or obesity, abdominal or obesity, morbid or obese or overweight* or over weight or body weight or adipose tissue or fatty tissue or indices body mass or body mass inde* or bmi or body fat or diabesity or diabetes-related obesity or obesity-related diabetes or intra-abdominal fat).ab.	949390
#3	Search: (T2DM or Diabetes mellitus non insulin dependent or type 2 diabetes or non insulin dependent diabetes mellitus or type ii diabetes or maturity onset diabetes or adult onset diabetes or diabetes mellitus type 2 or diabetes mellitus type ii or stable diabetes mellitus or T2dm or Niddm).ab.	210022
#2	Search: (intensification* or deintensification* or simplification* or titrations* or optimisation* or high-dose* insulin or low-dose* insulin or increase* dose or reduce* dose or decrease* dose or add* dose or total daily dose or dose* adjustment* or de-intensification*).ab.	75832
#1	Search: (insulin* or insulin,regular human or short-acting or rapid-acting or long-acting or intermediate-acting or NPH insulin or co-formulation or pre-mixed or basal bolus or ultra-long-acting or ultra-short-acting or multiple insulin injection or complex insulin or basal insulin or analogue* insulin or novolog or levemir or humalog or lantus).ab.	447227

Supplementary Table S5: Search details for EBSCO CINAHL

Date of search: 23 Feb 2026

Search	Query	Results
#5	Search: #1 AND #2 AND #3 AND #4 without filters	**399**
#4	Search: Obesity* OR Obesity,Morbid OR MH (Fat free mass) OR MH (Neoplasms,adipose tissue) OR MH (Adipose tissue distribution) OR XB (Abdominal fat) OR (Weights and measures) OR XB (Body weight) OR XB (Body weight changes) OR XB (high bmi or high body mass index or obesity) OR XB (overweight or obesity or obese or fat or unhealthy weight or high bmi)	489932
#3	Search: XB (diabetes type 2 or diabetes mellitus type 2 or diabetes 2) OR XB (diabetic patients or patients with diabetes or diabetes or diabetic) OR XB (adult-onset diabetes) OR XB (niddm) OR XB (type 2 diabetes or type 2 diabetes mellitus or t2dm) OR XB (high blood glucose) OR XB (uncontrolled diabetes) OR TI (diabetes type 2 or diabetes mellitus type 2 or diabetes 2)	245633
#2	Search:<br>XB (intensification) OR XB (Deintensification) OR XB (modification plan) OR XB (optimisation) OR XB (titration) OR MH (Maximum tolerated dose) OR MH (Dose-response relationship) OR XB (Total daily dose) OR MH (Dosage calculation) OR MH (Pharmacological and biological treatment) OR MH (Treatment switching)	44591
#1	Search: XB (insulin) OR XB (insulin aspart) OR XB (insulin lispro) OR XB (Insulin sensitivity) OR XB (Insulin resistance) OR XB (Insulin, intermediate-acting) OR XB (Insulin, short-acting) OR XB (Insulin, long-acting) OR XB (Insulin, rapid-acting) OR XB (Insulin infusion systems) OR XB (Insulin injection) OR XB (Biphasic insulins)	70451
